# A MoS_2_–MWCNT based fluorometric nanosensor for exosome detection and quantification†

**DOI:** 10.1039/c9na00248k

**Published:** 2019-06-19

**Authors:** Mahnoush Tayebi, Mohammad Tavakkoli Yaraki, Hui Ying Yang, Ye Ai

**Affiliations:** Pillar of Engineering Product Development, Singapore University of Technology and Design 8 Somapah Road Singapore 487372 Singapore aiye@sutd.edu.sg +65 6499 4553; Department of Chemical and Biomolecular Engineering, National University of Singapore 4 Engineering Drive 4 Singapore 117585 Singapore; Institute of Materials Research and Engineering, Agency for Science, Technology, and Research (A*STAR) 2 Fusionopolis Way 138634 Singapore

## Abstract

Circulating exosomes in body fluids are involved in many diseases and have important roles in pathophysiological processes. Specifically, they have emerged as a promising new class of biomarkers in cancer diagnosis and prognosis because of their high concentration and availability in a variety of biological fluids. The ability to quantitatively detect and characterize these nano-sized vesicles is crucial to make use of exosomes as a reliable biomarker for clinical applications. However, current methods are mostly technically challenging and time-consuming which prevents them from being adopted in clinical practice. In this work, we have developed a rapid sensitive platform for exosome detection and quantification by employing MoS_2_–multiwall carbon nanotubes as a fluorescence quenching material. This exosome biosensor shows a sensitive and selective biomarker detection. Using this MoS_2_–MWCNT based fluorometric nanosensor to analyze exosomes derived from MCF-7 breast cancer cells, we found that CD63 expression could be measured based on the retrieved fluorescence of the fluorophore with a good linear response range of 0–15% v/v. In addition, this nanosensing technique is able to quantify exosomes with different surface biomarker expressions and has revealed that exosomes secreted from MCF-7 breast cancer cells have a higher CD24 expression compared to CD63 and CD81.

## Abstract 2

Exosomes are nanosized extracellular vesicles containing molecular information from their parental cells. Detection and quantification of exosomes can offer a non-invasive method for an early diagnosis of diseases such as cancer.

## Graphical abstract

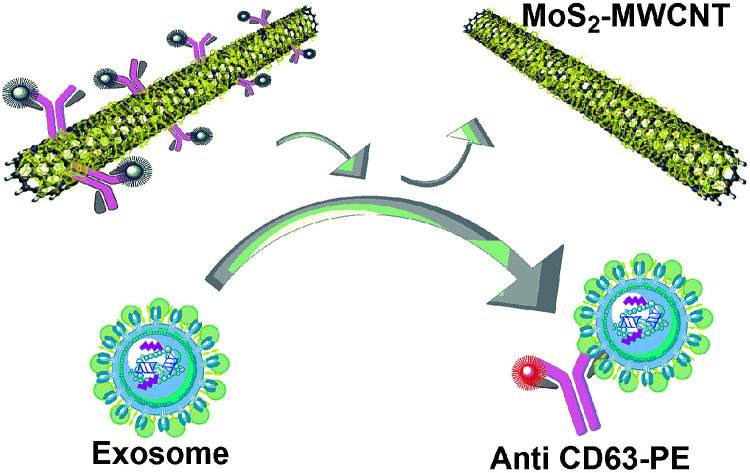

## 1. Introduction

Exosomes are nano-sized (30–150 nm), cup-shaped^1^ membrane-bound structures present inside large multivesicular endosomes^2^ and released by most eukaryotic cells.^3^ In recent years, extracellular vesicles (EVs), especially exosomes, have received increasing attention due to their cargo of protein, RNA, and DNA from their origin cell.^4^ It is currently believed that cells use EVs as a means of extracellular communication and exchange of proteins, lipids and nucleic acids.^5^ Recent studies further revealed that many biological fluids (including saliva, lymph, urine, milk, blood, synovial and amniotic fluids) contain vesicles which are released by early-stage tumors and carry various tumor markers.^6–8^ These micro- and nano-vesicles are potentially measurable in easily accessible body fluids and can be useful as a diagnostic and prognostic biomarker for early detection of many diseases and disorders, mainly, as essential indicators of the state and progression of cancer.^9^ According to previous studies, exosomes originating from tumors are an invaluable source of cancer biomarkers.^10^ The most common exosome marker target proteins are tetraspanins including CD9, CD63, and CD81. Tumor-associated markers are epithelial cell adhesion molecules (EpCAM), IGF-1R α units (α-IGF-1R), CA125, CD41b, and E-cadherin.^11–13^ In addition to tetraspanins and tumor associated markers, there are many other promising biomarkers which have important application in medical fields. For instance, CD24 is a biomarker expressed in breast cancer solid tumor and in hematological malignancies. The expression of this protein in ovarian cancer, non-small cell lung cancer, and prostate cancer has also been widely studied and it has been shown that CD24 is associated with an adverse prognosis in these cancers.^14,15^ In another study, it has been reported that there is a higher quantity of total exosomes in cancer patients compared with healthy volunteers as exosomes are secreted in larger amounts during carcinogenesis.^16^ Hence, achieving an accurate technique to analyze these small vesicles from a heterogeneous biological fluid which contains many other biomolecules with similar physical characteristics is essential for both diagnosis and therapy purposes.^17,18^ Biomarkers are commonly analyzed by using immunological methods such as enzyme linked immunosorbent assays (ELISA) or Western blot (WB) which are expensive and lengthy assays.^19^ Current protocols for exosome concentration measurement are still very semi-quantitative and mainly rely on procedures of total protein concentration or nanoparticle tracking analysis. In most of these current techniques, purification and isolation procedures such as ultracentrifugation are essential before concentration measurement which is a time-consuming process.^4,16^ Hence, rapid and sensitive detection of exosomes is still a challenge. Accordingly, a reliable and sensitive detection of exosomes originating from cancer cells is needed for early diagnosis and prognosis of cancer.^20,21^

Two-dimensional nanomaterials such as graphene oxide and transition metal dichalcogenides (TMDs) have been widely used as nano-quenchers in biomolecule detection in recent years.^22,23^ The mechanism of detection with these nano-quenchers is based on fluorescence resonance energy transfer (FRET), in which the fluorophore (donor) transfers energy to the nano-quencher (acceptor) and provides an “on–off” sensor to detect the biomolecules of interest.^24,25^ MoS_2_–MWCNT is a three-dimensional hierarchical nanostructure, which has one-dimensional MWCNT backbones with two-dimensional MoS_2_ nanosheets grown on the surface of MWCNTs and also possesses partially standing branch features.^26^ Preferably, the growth of MoS_2_ nanosheets on the outer layer of MWCNTs provides an even larger area for adsorbing the target molecule compared to other 2D materials.^27^

Based on immunoelectron microscopy studies, a variety of endocytic membranes have tetraspanin proteins (CD63, CD81, and CD9) which have been identified as the most abundant exosomal markers among various types of exosomes.^28,29^ It is therefore assumed that the number of CD63 positive exosomes represents their total number. Herein, we have developed a method using MoS_2_–MWCNT and the CD63 antibody to quantify the exosome concentration in biological samples. To the best of our knowledge, this is the first study to apply MoS_2_–MWCNT in exosome analysis using its capacity of biomolecule adsorbing and fluorescence quenching. CD63 protein as the most common biomarker in all types of exosomes is chosen to identify exosomes in this study. Fluorescently labeled anti-CD63 is adsorbed on the surface of MoS_2_–MWCNT; however their interaction is still not fully understood.^30^ The total exosome concentration was measured by using a nanoparticle tracking analysis (NTA) system. In addition, the application of this method for measuring the abundance of other types of exosomal biomarkers has been further studied to distinguish subpopulations of exosomes based on the concentration of biomarkers of interest.

## 2. Experimental section

### 2.1. Materials

Anti-human CD63 PE (R-phycoerythrin) and CD24 FITC (fluorescein isothiocyanate), monoclonal antibodies (Abs), were purchased from eBioscience, Inc. CD81 monoclonal antibody (M38) FITC and Total Exosome Isolation Reagent were obtained from Thermo Fisher Scientific, USA. L-MWCNT-60100 (Shenzhen Nanotech Port Co., Ltd) was used in the synthesis of a MoS_2_–MWCNT nanostructure according to previous work.^26,31^ Fluorescence spectra were recorded by using a Tecan microplate reader infinite M200 at the excitation wavelengths of 488 and 494 nm based on the used fluorophores. Phosphate buffered saline, 1*X* solution (Fisher Scientific, Inc.) and ultrapure water obtained from a Millipore filtration system were used as the dissolving solutions. All the reagents applied in the breast cancer cell (MCF-7) culture including Dulbecco's modification of Eagle's medium (DMEM), 10% fetal bovine serum (FBS), and antibiotics including penicillin and streptomycin were purchased from Thermo Fisher Scientific, USA and the MCF-7 cell line was purchased from the American Type Culture Collection (ATCC cat. no. HB-72). All NTA analyses were performed by using a ZetaView system, Particle Metrix GmbH.

### 2.2. Characterization

The morphology of the MoS_2_–MWCNT nanostructure and its absorbance spectrum were obtained by using a scanning electron microscope (JEOL JSM-7600F) and a UV-vis spectrophotometer (Tecan microplate reader infinite m200), respectively. A Zetasizer Nano Z system (Malvern Instruments Ltd.) was used to measure the zeta potential of the MoS_2_–MWCNT nanostructure.

### 2.3. MoS_2_–MWCNT synthesis

The solvothermal method was applied to synthesize the MoS_2_–MWCNT nanostructure according to the protocol developed in a previous study.^31^ Briefly, 220 mg (NH_4_)_2_MoS_4_ powder as the single reactant and 100 mg MWCNTs were mixed with 30 mL *N*,*N*-dimethylformamide (DMF) as the solvent. A uniformly dispersed solution was obtained after sonication and then placed in an autoclave (200 °C) for 10 hours to form MoS_2_–MWCNT composites. After purification and at least 5 times washing with DI water, the MoS_2_–MWCNT nanostructure was obtained with a Mo/C ratio of 1 : 5. The stock solution of MoS_2_–MWCNT is very stable and does not need to be maintained under special lab conditions. It was stored at room temperature and sonicated for one hour before each experiment.

### 2.4. Sample preparation

The MCF-7 cells were sub-cultured every 48 or 72 hours to obtain 80–90% confluence under 37 °C, 5% (v/v) CO_2_ in a humidified incubator. During sub-culturing, 0.25% trypsin–EDTA solution was used for dissociation of cell monolayers. After achieving 80–90% confluence, the cell culture supernatant was collected and centrifuged at 2000*g* for 30 min before filtering through a 0.22 μm filter. To establish an assay protocol for specific exosome detection and quantification, we first used a commercial total exosome isolation reagent according to the manufacture's protocol and purified the exosomes through a precipitation method. After precipitation of exosomes as a pellet, it was resuspended in PBS solution and was kept at 4 °C for immediate use, or at −20 °C for long-term storage. Then we characterized the isolated exosomes by using a NTA system to measure the concentration and size distribution.

To measure the quenching ability of the MoS_2_–MWCNT nanocomposites and obtain the optimum concentration of the nanoquencher, a series of different concentrations of MoS_2_–MWCNT nanocomposites were prepared in PBS solution and 2% v/v anti-CD63-PE (with 0.5 μg mL^−1^ final concentration) was added to each sample.

### 2.5. CD63 positive exosome detection

To demonstrate the positive detection of the CD63 exosome, the MoS_2_–MWCNT nanostructure was incubated with 2% v/v (0.5 μg mL^−1^) of anti-CD63-PE at room temperature in a dark environment. After 10 minutes, a series of concentrations of exosome solution from 5 to 800 v/v% were added to the quenched solution and incubated at room temperature for 60 minutes (the final volume of each sample was 50 μL, and the final concentration of the antibody was 0.1 μg mL^−1^). Then the samples were added to the individual wells on a black 384-well flat-bottomed microplate and recovered fluorescence of anti-CD63-PE was measured by using a microplate reader for each sample.

### 2.6. Specificity of exosome detection

The specificity of the developed exosome sensing technique was examined by measuring the relative fluorescence intensity recovery of anti-CD63-PE, anti-CD81-FITC, and anti-CD24-FITC when mixed with the MCF-7 cell culturing medium. The supernatant of the MCF-7 cell culturing medium was collected after 48 hours of incubation in a humidified incubator and was centrifuged at 2000*g* for 30 min to remove dead cells, large proteins and cellular debris as a pellet. Then, it was filtered through a 0.22 μm filter. Before adding the quenched anti-CD63-PE/MoS_2_–MWCNT complex, 500 μL of the total exosome isolation reagent was added to 1 mL of the centrifuged cell media and the exosomes were isolated according to the protocol provided by the manufacture. As the type and amount of dye in each dye-labelled antibody are different, the fluorescence recovery rate of the samples was measured with respect to the fluorescence of each dye-labelled antibody (0.1 μg mL^−1^ in PBS).

## 3. Results and discussion

**Fig. 1:**
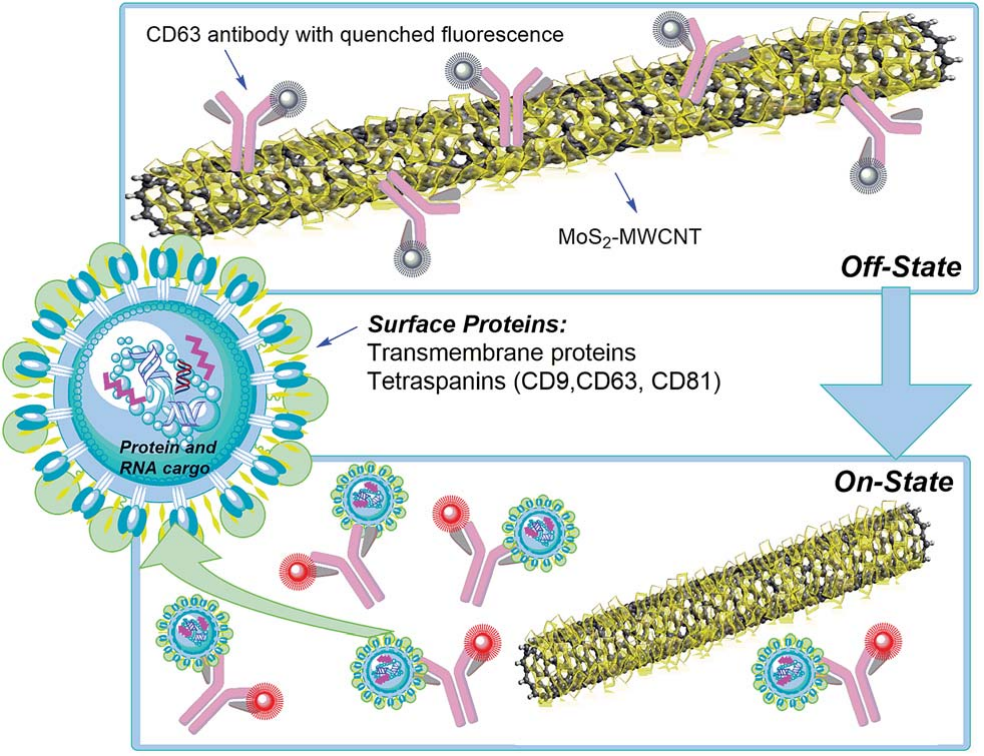
Schematic illustration of the MoS_2_–MWCNT based nano-sensor for exosome detection and quantification.

The proposed nano-sensor provides a rapid and sensitive technique to measure exosome concentration and could detect any biomarker of interest. Fig. 1 shows the MoS_2_–MWCNT based fluorescence sensing platform for exosome detection. Firstly, anti-CD63-PE is adsorbed on the surface of MoS_2_–MWCNT and its fluorescence is quenched off due to the fluorescence resonance energy transfer (FRET) from the PE-conjugated antibody to the MoS_2_–MWCNT nanostructure. After introducing exosomes into this mixture, due to the higher binding force between target exosomes and anti-CD63-PE, a detachment of anti-CD63-PE from MoS_2_–MWCNT causes a rapid fluorescence recovery. The morphology and absorbance spectra of the MoS_2_–MWCNT nanostructure are shown in Fig. S1 and S2,† respectively. MoS_2_ has a layered structure extended out of cylindrical tubules and provides a much larger surface area to adsorb anti-CD63-PE. It also shows a broad absorbance spectrum from the UV to NIR region. According to the Beer–Lambert law *A* = (*α* × *L*) × *C*, where *A* is the measured absorbance, *α* is the extinction coefficient, *L* is the path length and *C* is the concentration. The extinction coefficient was calculated to be 102.31 L g^−1^ cm^−1^ which is a much higher value compared with that of previously reported 2D layered materials.^32,33^

**Fig. 2:**
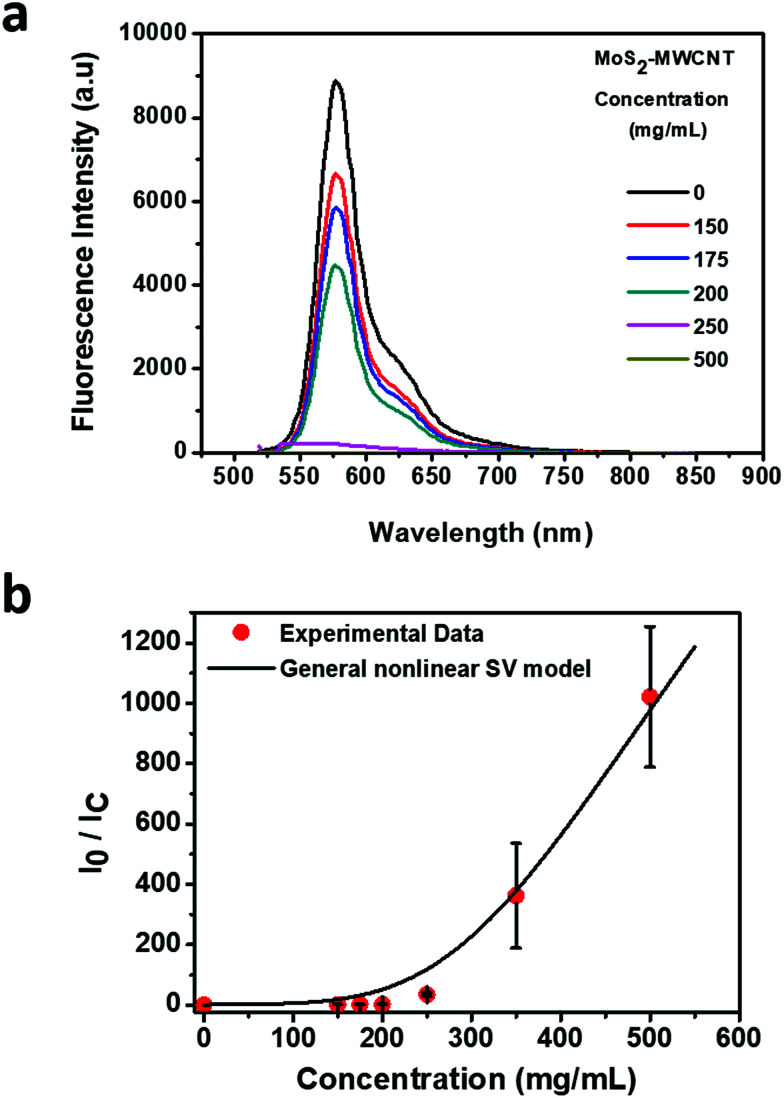
Quenchability of MoS_2_–MWCNT nanostructures. (a) Quenchability measurement with different concentrations of MoS_2_–MWCNT (150, 175, 200, 250, 350 and 500 mg mL^−1^). (b) Maximum fluorescence intensity as a function of MoS_2_–MWCNT concentration. The antibody concentration is 0.1 μg mL^−1^.

Based on the absorbance measurement of six different MoS_2_–MWCNT concentrations, 500 mg mL^−1^ shows the highest absorbance value. In addition, according to the quenchability data (Fig. 2a and b), the MoS_2_–MWCNT sensor shows the total quenching of the fluorescence at this concentration, which confirms that the optimum concentration of MoS_2_–MWCNT is 500 mg mL^−1^. To assess the quenching ability of MoS_2_–MWCNT, the quenching fluorescence data (*I*_0_/*I*_C_) are plotted *versus* the concentration of MoS_2_–MWCNT (Fig. 2b). As can be seen, the fluorescence quenching data have a nonlinear trend. Therefore, the traditional linear form of the Stern–Volmer (SV) model could not be applied to describe the quenching ability of the MoS_2_–MWCNT nanostructure as a quencher for anti-CD63-PE. The positive deviation at a high concentration of the quencher could be attributed to the simultaneous presence of dynamic (collisional) and static quenching.^34,35^ Chen *et al.*^36^ have developed a general nonlinear SV model based on the Sips adsorption isotherm model^37^ to describe the fluorescence quenching with nonlinear trends that could be written as the following equation:1
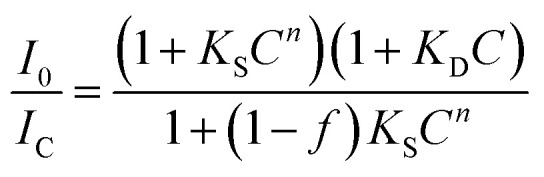
where *C* is the concentration of the quencher, and *I*_0_ and *I*_C_ are the fluorescence intensity in the absence and presence of the quencher, respectively. *K*_S_ and *K*_D_ are the static and dynamic quenching constants, respectively. The exponent *n* refers to the effective adsorption sites, adsorption strength or the index of affinity heterogeneity. The parameter *f* refers to the fractional accessible sites for the fluorophore in the quencher, which should be between 0 and 1. Table S1† presents the parameters of the general nonlinear SV model for the data shown in Fig. 2b. The near unity value for parameter *f* indicates the efficient quenching ability of MoS_2_–MWCNT. This parameter is in good agreement with a high *n* value (3.474), which could be attributed to the high affinity of anti-CD63-PE to MoS_2_–MWCNT as a quencher. Furthermore, the combination of static and dynamic quenching, giving rise to the nonlinear quenching behavior of MoS_2_–MWCNT, could be attributed to the non-uniform surface with inequivalent adsorption sites existing in MoS_2_–MWCNT. To confirm the adsorption of anti-CD63-PE on the surface of the MoS_2_–MWCNT nanostructure, the zeta-potential of MoS_2_–MWCNT was measured before and after addition of the antibody (Fig. S3†). The substantial change in the zeta-potential from −30 mV to −13 mV after the addition of the antibody shows that it is successfully attached to the MoS_2_–MWCNT nanostructure.

**Fig. 3:**
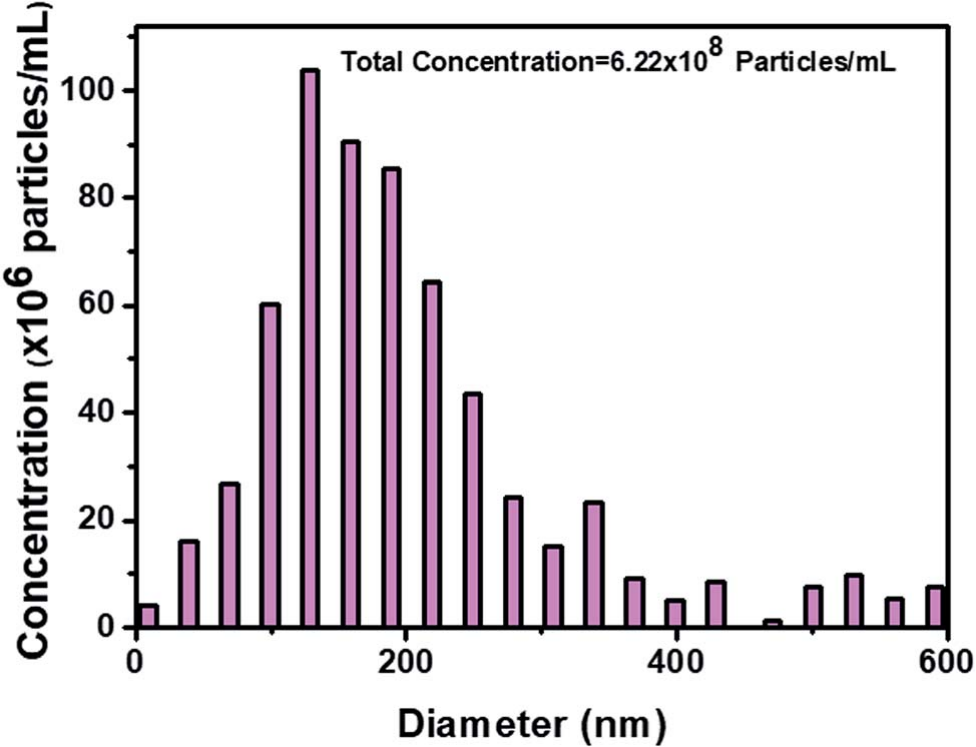
Concentration and size distribution of exosomes after purification with a commercial exosome isolation kit.

**Fig. 4:**
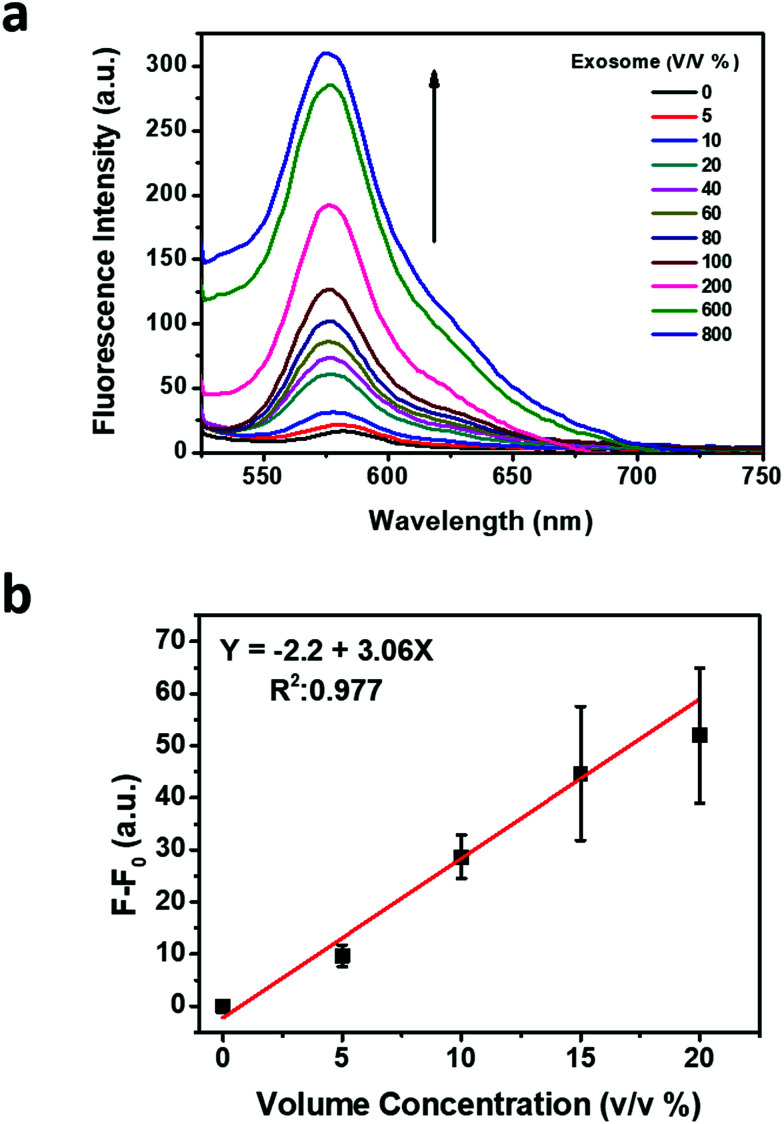
Exosome detection and concentration quantification based on the fluorescence recovery. (a) Fluorescence spectra of anti-CD63-PE after incubation with a series of volume percentages of exosomes. (b) Calibration curve according to the recovered fluorescence intensity.

The original concentration and size distribution of exosomes were measured by using nanoparticle tracking analysis (NTA), which uses the Brownian motion of particles in liquid and characterizes exosomes based on their size and concentration (Fig. 3). The recovered fluorescence intensity indicates the increase of recovered fluorescence by increasing the concentration of the exosome (Fig. 4a). The calibration curve of recovered fluorescence *versus* concentration of the exosome is shown in Fig. 4b which exhibits a linear region between 0 and 15 v/v% (0–11.1 × 10^6^ particles per mL) concentrations with a detection limit of 2.0408 v/v% (14.8 × 10^5^ particles per mL) based on the 3σ rule. Cancer derived exosomes in body fluids are available in high concentrations (10^9^–10^12^ exosomes per mL in blood),^38^ which are comparable to normal exosome concentration (10^11^ exosomes per mL in blood).^8^ It is thus conceivable that the application of MoS_2_–MWCNT to exosome detection provides an assay with a sufficient detection sensitivity.

**Fig. 5:**
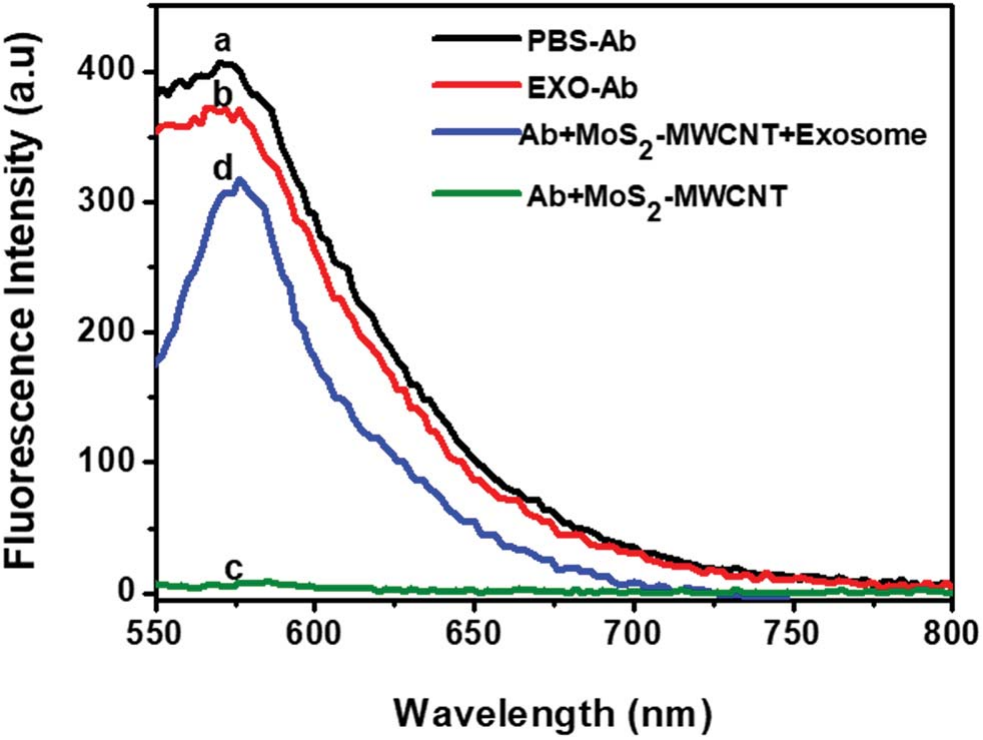
Fluorescence intensity of different components of the sensor. (a) and (b) show the fluorescence of anti-CD63-PE in the absence of the MoS_2_–MWCNT nanoquencher. (c) shows the quenched fluorescence in the presence of the nanoquencher and (d) shows the retrieved fluorescence after adding the exosome sample (concentration of the antibody is the same in all samples). It shows that the fluorescence quenching and recovery are just due to the presence of MoS_2_–MWCNT and exosomes respectively.

To demonstrate the mechanism of exosome sensing *via* the MoS_2_–MWCNT sensor, the fluorescence spectrum of anti-CD63-PE alone and in the presence of an exosome was recorded (Fig. 5, curves a and b). It showed a strong fluorescence emission in both measurements (peak at 575 nm) owing to the presence of PE (R-phycoerythrin), while upon the addition of the nano-quencher, the fluorescence intensity of anti-CD63-PE dropped significantly, which indicates that most of the anti-CD63-PE was adsorbed on the surface of MoS_2_–MWCNT and caused the adequate quenching of the fluorescence (Fig. 5, curve c). However, after adding the target exosome, the fluorescence was recovered considerably. This trend shows a high binding rate of anti-CD63-PE and exosomes which prevents placing the PE fluorophore at close distances to the nano-quencher and stops FRET occurring between them (Fig. 5, curve d).

**Fig. 6:**
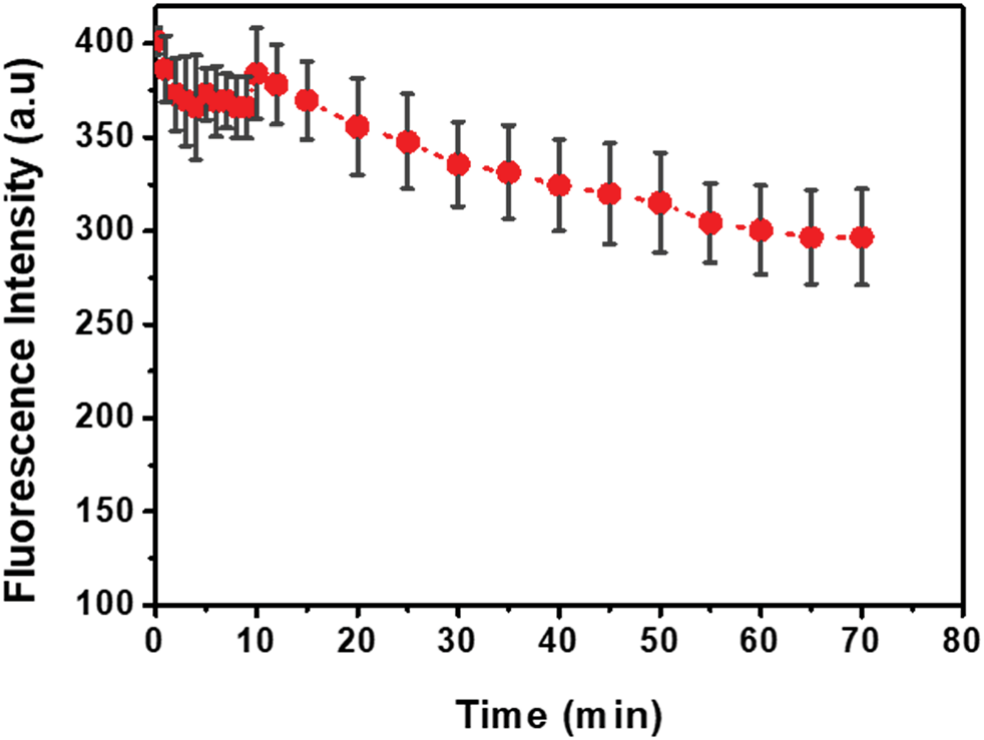
Fluorescence intensity recovery kinetic. Anti-CD63-PE and MoS_2_–MWCNT concentrations are 0.1 ug mL^−1^ and 500 mg mL^−1^, respectively.

The kinetic behavior of anti-CD63-PE and exosome hybridization was studied by monitoring the recovery of fluorescence intensity in 60 min after introducing exosome solution into the quenched anti-CD63-PE/MoS_2_–MWCNT complex (Fig. 6). The preparation time for the quenched complex was less than 5 minutes and fluorescence recovered very fast in a few seconds, once the exosome solution was added to the quenched complex. However, the fluorescence intensity was not stable in the first 10 minutes, which could be due to some false interactions between exosomes and anti-CD63-PE. Based on this figure, after one hour, fluorescence was more stable which demonstrates a specific and stable binding to the antigen. Hence, all the fluorescence measurements in this study were also done after one hour to achieve a stable fluorescence intensity. Table S2† presents a comparison of the reported results for the LOD and detection time of exosomes *via* different methods. The results indicate that the developed fluorometric method in this study has an acceptable LOD as well as detection time, and can compete with other methods due to its simple procedure and low equipment cost.

**Fig. 7:**
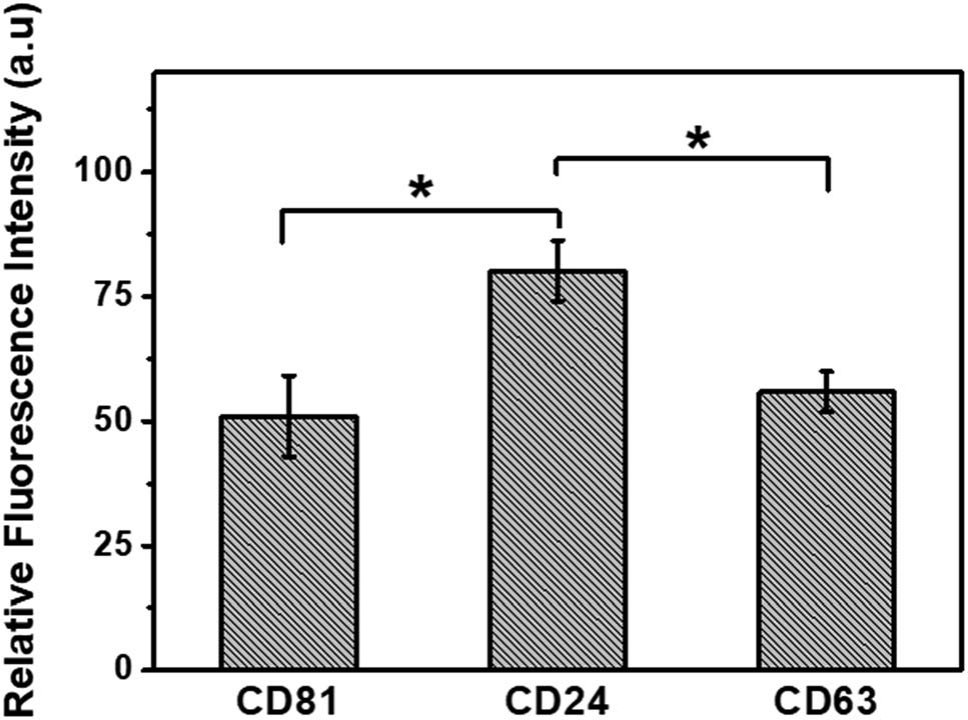
Relative fluorescence recovery of FITC conjugated anti-CD81, anti-CD24 and PE conjugated anti-CD63, where the relative fluorescence refers to *F*/*F*_c_, and *F*_c_ is the fluorescence intensity of FITC conjugated and PE conjugated control antibodies at *λ* = 520 and 575 nm, respectively.

For testing the specificity of this sensor, fluorescent dye-labeled anti-CD63, CD81, and CD24 were used as the sensing agents for the detection of specific exosomes derived from MCF-7 cells. According to Fig. 7, the fluorescence recovery of CD24 is much higher than that of CD63 and CD81, and it is in good agreement with previous studies which have reported an enhancement in the concentration of CD24 protein in cancer cells.^6,15^ It is therefore concluded that this detection platform is highly sensitive to the concentration of biomarkers at different expression levels and has a great potential application in the detection of specific biomarkers for diseases.

## 4. Conclusions

In this study, we developed an easy, fast and efficient sensing platform to detect exosomes with specific biomarkers using MoS_2_–MWCNT nanocomposites. This fluorescence-based biosensor has a linear range of 0–15 v/v% (0–11.1 × 10^6^ particles per mL) with a detection limit of 2.0408 v/v% (14.8 × 10^5^ particles per mL) which is lower than the exosome concentration range in body fluids^8^ and is comparable to other methods (*e.g.*, 5 × 10^7^ particles per mL ^39^ and 2.2 × 10^7^ exosomes per mL ^8^). Also, we tested the specificity of this sensor which showed a high selectivity for the biomarkers of interest. The robustness of the biosensor provides a mechanism to measure different biomarkers upon choosing the related antibodies. It is therefore not limited to detect only anti-CD63 and has the potential to measure any other biomarker. Furthermore, this technique could be a potential platform for *in situ* detection of exosomes for early detection and therapy of cancer.

## Conflicts of interest

There are no conflicts to declare.

## Supplementary Material

NA-001-C9NA00248K-s001
