# Supplementary material for: A MoS2–MWCNT based fluorometric nanosensor for exosome detection and quantification
Source: Nanoscale Adv. 2019 Jun 19;1(8):2866–72. doi: 10.1039/c9na00248k (PMC9419881; doi:10.1039/c9na00248k)
Supplement: NA-001-C9NA00248K-s001 [file NA-001-C9NA00248K-s001.pdf]

Electronic Supplementary Material (ESI) for Nanoscale Advances.
This journal is © The Royal Society of Chemistry 2019

Supplementary Information

MoS_2-MWCNT based Fluorometric Nanosensor for Exosome Detection and Quantification

Mahnoush Tayebi [a], Mohammad Tavakkoli Yaraki [b,c], Hui Ying Yang [a] and Ye Ai [a,*]

[a] Pillar of Engineering Product Development, Singapore University of Technology and Design, 8 Somapah Road, Singapore 487372, Singapore

[b] Department of Chemical and Biomolecular Engineering, National University of Singapore, 4 Engineering Drive 4, Singapore 117585, Singapore

[c] Institute of Materials Research and Engineering, Agency for Science, Technology, and Research (A*STAR), 2 Fusionopolis Way, 138634, Singapore

* Corresponding author. Email: aiye@sutd.edu.sg; Tel: (+65) 6499 4553

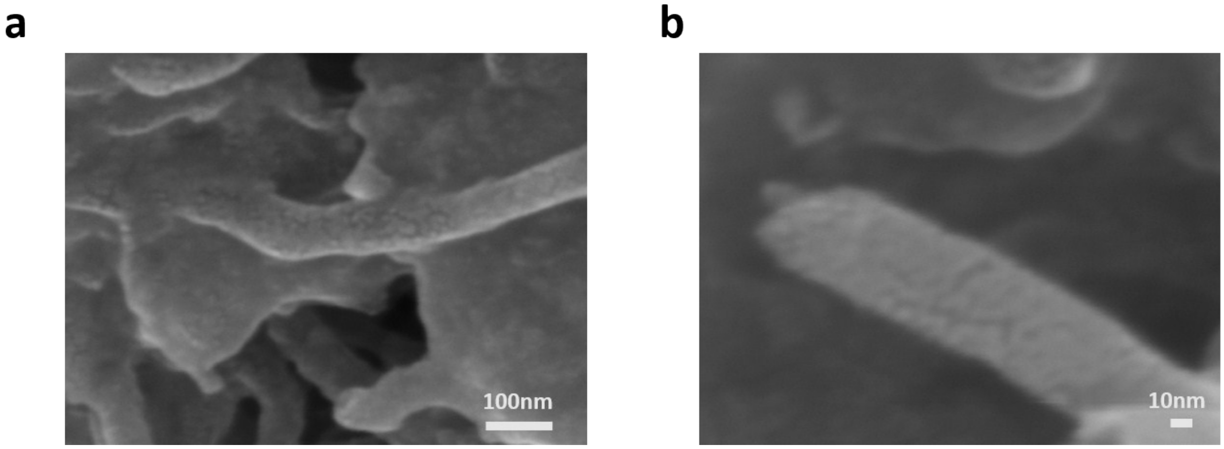

Fig. S1 Scanning electron microscopy of MoS_2-MWCNT with different magnification in (a) and (b).

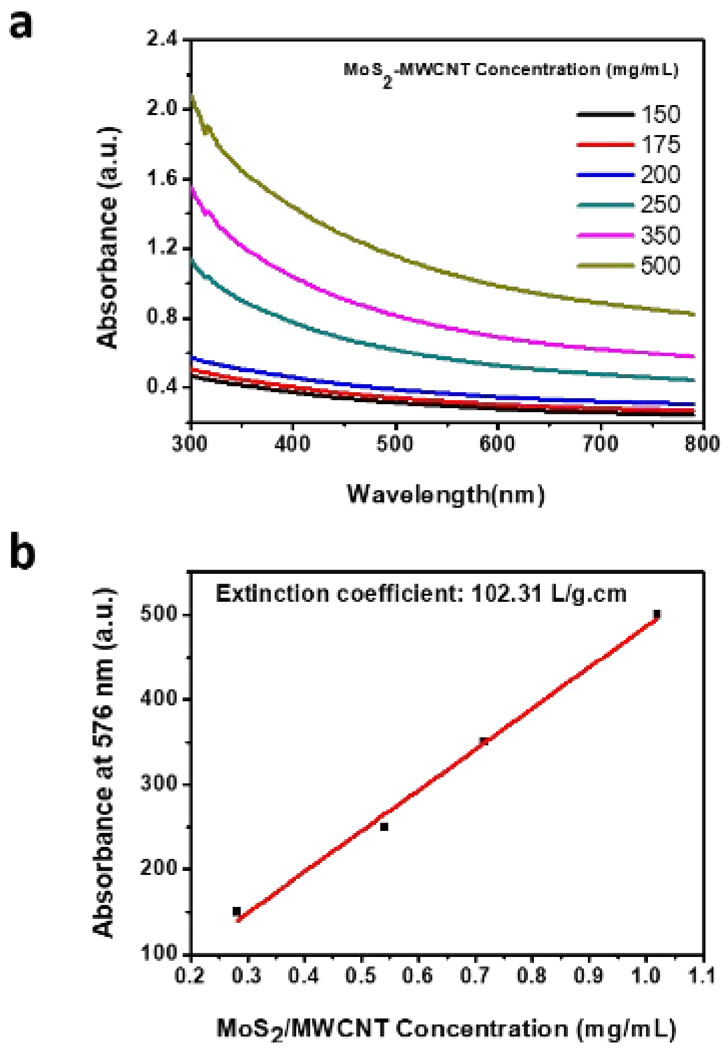

Fig. S2 Absorbance of different concentrations of MoS_2-MWCNT with a broad absorption band from UV to NIR region, which is a good candidate for PE (R-phycoerythrin) with emission wavelength of 575 nm.

Table S1.

The parameters of general nonlinear Stern-Volmer model for fluorescence quenching of anti-CD63-PE in the presence of MoS_2-MWCNT as a quencher

K_S (mL/mg)	K_D (mL/mg)	f	n	R^2
7.724×10^{-8}	0.02743	0.9903	3.474	0.9852

Table S2

Comparison of the Limit of Detection (LOD) and Detection Time of Different Methods for Exosome Determination

Detection Method	Purification Method	Detection Time	Limit of Detection	Method Limitation	Reference
Anodic stripping voltammetric quantification	Total exosome isolation reagent and CD63 antibody-functionalised magnetic beads	>1hr	10^5 exosomes/mL	Not given	[1]
Aptamer based electrochemical biosensor	ExoQuick-TC exosome precipitation reagent	83 min	10^6 exosomes/mL	Not given	[2]
Colorimetric (mimicking peroxidase ability of single-wall carbon nanotubes)	Ultracentrifugation	40 min	5.2×10^8 exosomes/mL	Susceptible to interference due to developing a “signal-on” strategy to replace “signal-off” strategy	[3]
Electrochemical	Total exosome isolation reagent	60 min	4.7×10^8 exosomes/mL	Not given	[4]
Nanostructured herringbone chip combined with a sandwich exosome enzyme-linked immunosorbent assay (ELISA)	Exosome capture on the antibody modified chips/ ultracentrifugation	-	10^4 exosomes/mL	Limited preparative sample processing capacity for bulk exosomal content analysis	[5]
Microfluidic and fluorescence	immobilizing vesicles in a microfluidic device (ExoChip)	70 min	0.5 pM	Not given	[6]
Electrochemical sandwich immunosensor	Ultracentrifugation	60 min	2×10^5 exosomes/mL	Long incubation times and multi-steps process	[7]
Fluorescence	Total exosome isolation reagent	60 min	14.8×10^5 exosomes/mL	Susceptible to influence from nonspecific interactions of antibodies	This work

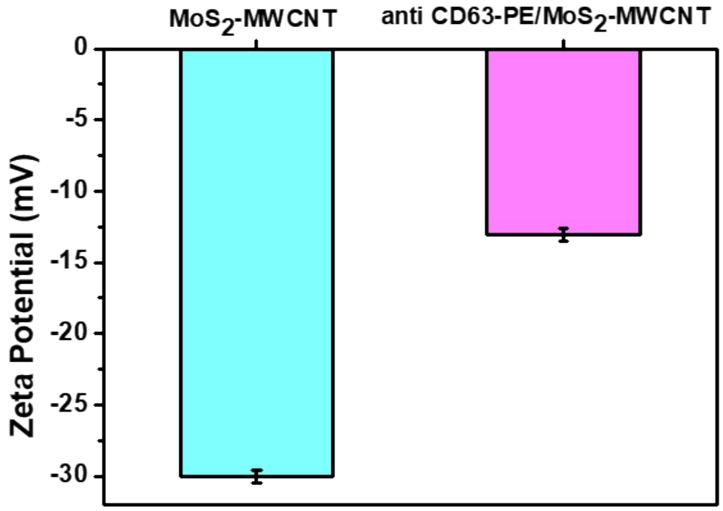

Fig. S3 Zeta potential of MoS_2-MWCNT nanocomposites (a) before and (b) after adding anti CD63-PE, which shows a significant decrease in the zeta potential value and confirms the adsorption of anti CD63-PE on MoS_2-MWCNT.

References

1. Boriachek, K.; Islam, M. N.; Gopalan, V.; Lam, A. K.; Nguyen, N.-T.; Shiddiky, M. J. A., Quantum dot-based sensitive detection of disease specific exosome in serum. *Analyst* **2017,** *142* (12), 2211-2219.
2. Zhou, Q.; Rahimian, A.; Son, K.; Shin, D.-S.; Patel, T.; Revzin, A., Development of an aptasensor for electrochemical detection of exosomes. *Methods* **2016,** *97*, 88-93.
3. Xia, Y.; Liu, M.; Wang, L.; Yan, A.; He, W.; Chen, M.; Lan, J.; Xu, J.; Guan, L.; Chen, J., A visible and colorimetric aptasensor based on DNA-capped single-walled carbon nanotubes for detection of exosomes. *Biosensors and Bioelectronics* **2017,** *92*, 8-15.
4. Yadav, S.; Boriachek, K.; Islam, M. N.; Lobb, R.; Möller, A.; Hill, M. M.; Hossain, M. S. A.; Nguyen, N. T.; Shiddiky, M. J., An Electrochemical Method for the Detection of Disease-Specific Exosomes. *ChemElectroChem* **2017,** *4* (4), 967-971.
5. Zhang, P.; Zhou, X.; He, M.; Shang, Y.; Tetlow, A. L.; Godwin, A. K.; Zeng, Y., Ultrasensitive detection of circulating exosomes with a 3D-nanopatterned microfluidic chip. *Nature Biomedical Engineering* **2019**, 1.
6. Kanwar, S. S.; Dunlay, C. J.; Simeone, D. M.; Nagrath, S., Microfluidic device (ExoChip) for on-chip isolation, quantification and characterization of circulating exosomes. *Lab on a Chip* **2014,** *14* (11), 1891-1900.
7. Doldán, X.; Fagúndez, P.; Cayota, A.; Laíz, J.; Tosar, J. P., Electrochemical Sandwich Immunosensor for Determination of Exosomes Based on Surface Marker-Mediated Signal Amplification. *Analytical Chemistry* **2016,** *88* (21), 10466-10473.
